# The role of environment, dispersal and competition in explaining reduced co-occurrence among related species

**DOI:** 10.1371/journal.pone.0185493

**Published:** 2017-11-03

**Authors:** Ben G. Weinstein, Catherine H. Graham, Juan Luis Parra

**Affiliations:** 1 Dept. of Ecology and Evolution, Stony Brook University, Stony Brook, New York; United States of America; 2 Swiss Federal Research Institute (WSL), Birmensdorf, Zurich, Switzerland; 3 Instituto de Biología, Universidad de Antioquia, Medellín, Colombia; Universitat Trier, GERMANY

## Abstract

The composition of ecological assemblages depends on a variety of factors including environmental filtering, biotic interactions and dispersal limitation. By evaluating the phylogenetic pattern of assemblages, we gain insight into the relative contribution of these mechanisms to generating observed assemblages. We address some limitations in the field of community phylogenetics by using simulations, biologically relevant null models, and cost distance analysis to evaluate simultaneous mechanisms leading to observed patterns of co-occurrence. Building from past studies of phylogenetic community structure, we applied our approach to hummingbird assemblages in the Northern Andes. We compared the relationship between relatedness and co-occurrence among predicted assemblages, based on estimates of suitable habitat and dispersal limitation, and observed assemblages. Hummingbird co-occurrence peaked at intermediate relatedness and decreased when a closely-related species was present. This result was most similar to simulations that included simultaneous effects of phylogenetic conservatism and repulsion. In addition, we found older sister taxa were only weakly more separated by geographic barriers, suggesting that time since dispersal is unlikely to be the sole factor influencing co-occurrence of closely related species. Our analysis highlights the role of multiple mechanisms acting simultaneously, and provides a hypothesis for the potential importance of competition at regional scales.

## Introduction

The composition of ecological assemblages depends on diversification of clades in a region, colonization of species from outside that region and the niche overlap among species. When species diverge from a common ancestor, they tend to have a similar set of biotic and abiotic requirements, such that their niches are conserved [1,2]. Niche conservatism can result in co-occurrence of closely related species in local assemblages if conserved traits permit existence in a given environment (i.e environmental filtering)[3]. However, co-occurring related species may compete for limited resources due to shared functional morphology or foraging strategies [4,5]. Limiting similarity predicts that if niche overlap between two species surpasses some threshold, interspecific competition will exceed intraspecific competition, leading to local extinction of one competitor [6,7]. In addition, species may not co-occur because they have not dispersed to all suitable habitats, either because of insufficient time for dispersal or insurmountable geographic barriers [8,9].

A common approach for integrating evolutionary history and community ecology is to compare the phylogenetic structure of observed assemblages to a biogeographic species pool using randomization tests [10,11]. This approach has been increasingly criticized with several recent calls for more critical use of these methods [12–14]. Given the complexity of biogeographic patterns, and the potential influence of many mechanisms leading to similar patterns, it is often difficult to disentangle all possible causes in a single model.

Here we explore patterns of hummingbird co-occurrence and relatedness in the Northern Andes combining multiple analyses including: simulations, hierarchical models of co-occurrence and relatedness, predicted assemblages based on broad-scale environment, and analysis of habitat connectivity.

We simulated assemblages to simultaneously evaluate how different mechanisms influence the phylogenetic structure of assemblages. Simulated assemblages were created based on two mechanisms: phylogenetic conservatism of traits important to environmental filtering, and phylogenetic repulsion based on traits mediating competition [15,16]. Phylogenetic conservatism implies that the traits associated with species occurrence evolved with greater phylogenetic signal than by Brownian Motion, such that closely related species tend to co-occur given strong environmental filtering for the traits in question [1]. Phylogenetic repulsion implies that traits associated with species occurrence evolved with divergent selection, such that closely related species are less likely to co-occur if the traits in question lead to increased niche overlap resulting in increased competition. These simulations provide a basis of comparison as to how the simultaneous effects of phylogenetic conservatism and repulsion might influence patterns of phylogenetic co-occurrence in our empirical data.

We used a non-linear hierarchical model to simultaneously evaluate the effect of filtering and competition on patterns of co-occurrence, while allowing for species-level variation. This approach should improve upon previous studies that use randomization tests to identify assemblages as either underdispersed, overdispersed, or indistinguishable from a null expectation. Further, by using a hierarchical model we can evaluate the overall effect of relatedness on patterns of co-occurrence, while allowing for species-level variation. Rather than fitting a model to either an entire assemblage or species individually, a hierarchical model allows data-poor species to borrow strength from more commonly sampled species by allowing variance to be shared among all hummingbirds. This approach allows us to evaluate the shape of the relatedness and co-occurrence curve for each species and compare it to the curve based on predicted environmental suitability. In contrast, randomizations treat assemblages as the fundamental unit which implicitly assumes that all co-occurring species are influenced by the same mechanisms [17,18].

Evaluation of the phylogenetic structure of an assemblage requires a potential pool of species that could occur in a given location. While past studies have focused largely on geography in constructing species pools, we include information on environmental tolerances and dispersal limitation [19,20]. We used ensemble bioclimatic models based on current species distribution to evaluate environmental tolerances and included a dispersal filter based on elevational barriers between assemblages. We then compared these predicted assemblages to the observed assemblages based on the frequency of co-occurrence among related species. Further, we evaluated the possibility that early branching sister taxa are more likely to co-occur than late branching sister taxa due to increased time for dispersal across geographic barriers [21].

To explore our integrated approach, we used hummingbird assemblages from the Northern Andes. This system is relatively well-developed, with community phylogenetic studies finding support for both competition and environmental filtering as potential mechanisms influencing assemblage structure. Initial work using geographic species pools found evidence for phylogenetic clustering, which was attributed to environmental filtering in high elevation assemblages [22,23]. Acknowledging the importance of considering multiple mechanisms, subsequent analyses explored subsets of data including: sub-clades [24,25], environmentally restricted assemblages [26,27] and geographically constrained null models [28]. This most recent analysis revealed that once species pools were constrained by mean annual temperature, many assemblages were more evenly phylogenetically dispersed than previously appreciated. Our approach builds from this body of work by 1) explicitly accounting for environment using ensemble bioclimatic models, rather than single variables (as in [28]); 2) focusing on individual species, rather than assemblages, to evaluate phylogenetic structure; 3) moving beyond a dichotomous null-modelling framework that evaluates biotic interactions and environmental as opposing forces; and 4) comparing predictions of relatedness and co-occurrence to simulations built with known patterns of trait evolution. We hope that these improvements will provide increased rigor to the field of community phylogenetics as the community continues to evaluate what can be learned from pattern-based studies.

## Methods

### Simulation study to evaluate multiple mechanisms

**Fig 1:**
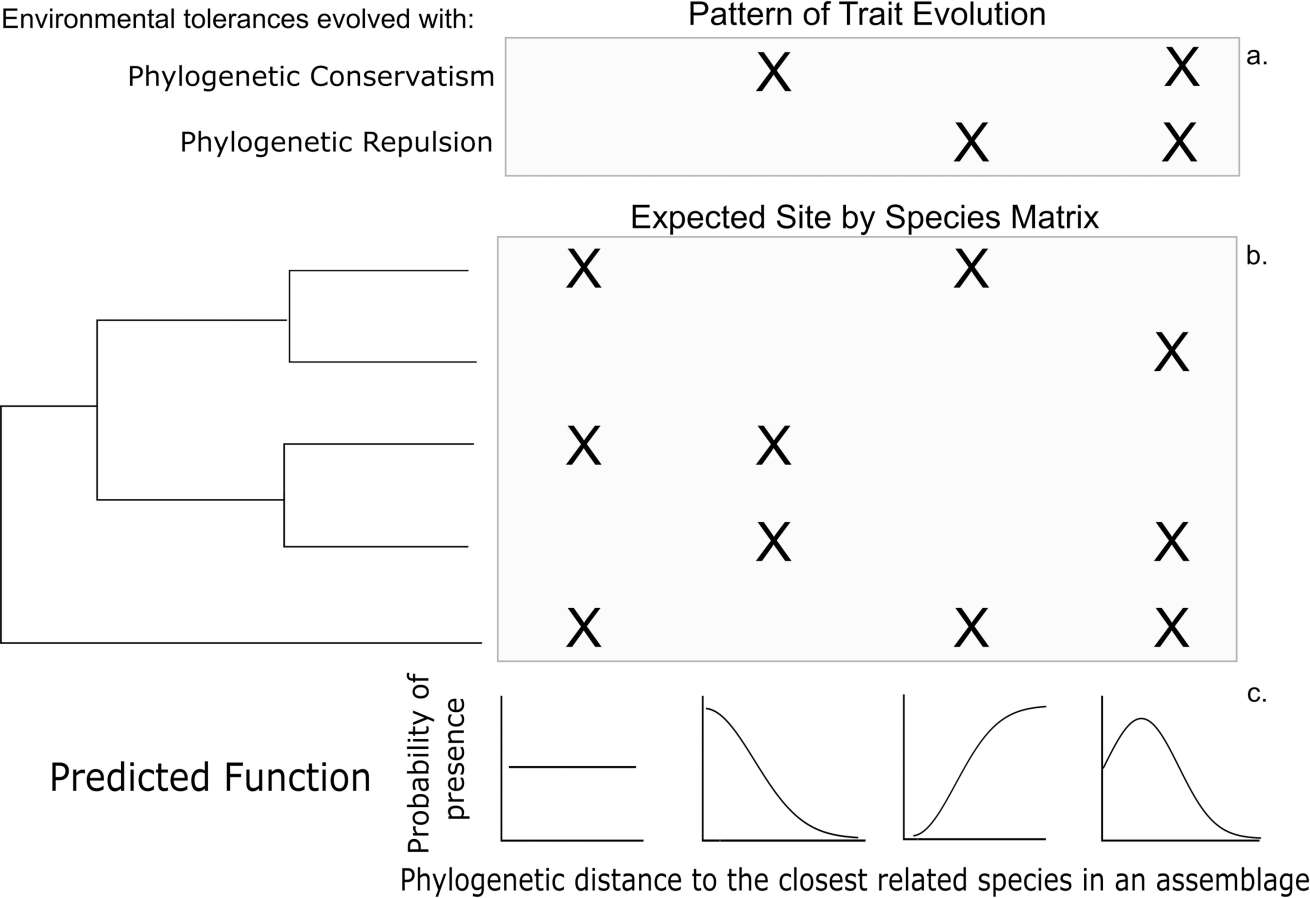
Conceptual diagram of four simulated assemblage types given known patterns of trait evolution. Each combination of phylogenetic conservatism and repulsion in the occurrence trait (a) would lead to a different pattern of co-occurrence among related species (b) leading to a different function representing the relationship between probability of presence and phylogenetic distance to the closest related species (c).

To determine the patterns of relatedness and co-occurrence as a function of known mechanisms, we created a range of scenarios for species co-occurrence and phylogenetic relatedness based on the degree of environmental filtering and limiting similarity (Fig 1). These scenarios were generated by evolving hypothetical species using varying degrees of phylogenetic conservatism and repulsion in environmental tolerances (following [29], using the 'scape' function in R packge PEZ, [30]) (Table 1). As phylogenetic conservatism increases, related species tend to co-occur more often. As phylogenetic repulsion increases, related species tend to co-occur less often. We calculated the relationship between phylogenetic distance to the closest related species and the probability of co-occurrence. Each simulation was repeated fifty times with a 10X10 square landscape with two environmental gradients and a balanced phylogeny with 16 taxa.

**Table 1:** Simulations used to evaluate the importance of environmental filtering and competition in structuring assemblages. G parameter is the strength of phylogenetic conservatism (g.center) and the strength of phylogenetic repulsion (g.repulse) as implemented in the R package PEZ [29]. When g.center = 0, the environmental tolerances evolve according to Brownian motion.

Simulation	G parameter (pez::scape)	Ecological Motivation
**No phylogenetic conservatism or repulsion**	g.center(0), g.repulse(0)	Neither environmental filtering nor competion is related to phylogenetically conserved traits. Co-occurrence is unrelated to relatedness.
**No phylogenetic conservatism, strong repulsion**	g.center(0), g.repulse(5)	The environment does not filter for phylogenetically conserved traits. However, competition among species is important. Co-occurrence weakly decreases with releatedness.
**Strong phylogenetic conservatism, no repulsion**	g.center(5), g.repulse(0)	The environment filters for a traits which are phylogenetically conserved. Competition does not limit co-occurrence. Co-occurrence will sharply increase with relatedness.
**Strong phylogenetic conservatism and strong repulsion**	g.center(5), g.repulse(5)	The environment filters for a traits which are phylogenetically conserved, but competition among closely related species limits niche overlap. Co-occurrence increases with relatedness until a threshold of niche overlap.

### Hummingbird and climate data

We used 230 hummingbird assemblage lists (presence/absence data) from published references, focusing on the Columbian and Ecuadorian Andes and adjacent lowlands [23,27]. These list included 133 species, with a mean occupancy of 18 sites. We excluded lists from eco-lodges with high density of hummingbird feeders and merged assemblages within 0.1° cells (~10km) because assemblages in close proximity may be non-independent. For the bioclimatic models, hummingbird point localities were collected from published literature, environmental organizations, and museum specimens [23,27]. To avoid circularity in predicted species presence, records from the assemblage data were not included in the localities used to create the bioclimatic models, and species with fewer than 20 occurrence records were removed from the analysis [31]. In total we had 22,046 records for 132 species, with an average of 166 localities per species. The phylogenetic relatedness to the closest related species was computed using the cophenetic distance in a regional phylogeny that included all 130 hummingbird species in the observed assemblages [32].

For climate covariates, we used the Worldclim database [33] at the 0.5 minute resolution (0.0083 degrees) and aggregated to 0.1 degrees to match the resolution of our assemblage data using the function ‘aggregate’ in the R package ‘raster’ [34]. We choose this relatively coarse resolution because we were interested in the broad climate drivers of distributions. While we acknowledge climate layers at this resolution for the Andes Mountains do not reflect climate variation along its steep environmental gradients, they should provide the general climate envelope we required for this study. Predicted habitat suitability does not automatically imply presence, rather we use the bioclimatic models to gauge the environmental suitability of a geographic location for each species assuming no dispersal limitation. We used principal component analysis and Pearson’s correlations on all 19 Worldclim variables to identify the following relatively non-correlated variables defining the environmental gradients in our region: mean annual temperature, mean annual precipitation, temperature seasonality (standard deviation of temperature across 12 months) and precipitation seasonality. These variables were highly correlated with the remaining 15 bioclimatic variables, but had correlation coefficients below 0.4 among each other. In addition, these variables have a biological basis and have been shown to predict hummingbird distributions in previous work [28,35,36]. Mean annual temperature is correlated with elevation and represents both energetic and flight (i.e., air density) constraints on hummingbirds [37]. Precipitation relates to the nectar availability and floral diversity of hummingbird-visited plants [38]. Temperature and precipitation seasonality broadly define important gradients in the Ecuadorian Andes ranging from dry Tumbesian regions in the southwest to the wet tropical forest in Amazonia.

### Ensemble bioclimatic models

To predict habitat suitability, we built ensemble models using maximum entropy (MAXENT), boosted regression trees (GBM), and generalized linear models (GLM) (R package biomod2 [39]). We used ensemble bioclimatic models because they combine results from multiple algorithms and tend to perform better than individual techniques, since they circumvent any idiosyncratic weakness of each modeling method [40]. The GLM allowed for quadratic terms with no interactions among environmental variables. We chose MAXENT because it performed well in comparisons with other presence-only data approaches [41], but see [42], and in previous work in the Andes [43–45]. GBM is a machine-learning method that estimates the relationship between a response variable and its predictors without *a priori* specification of a model by using cross-validation of smaller candidate trees [46]. For our GBM model, we used 2500 maximum trees with three-fold cross-validation. Output of all models scale from 0 (not suitable) to 1 (completely suitable) making it possible to generate an ensemble model (see below). For all modeling methods we used 2000 background points for each model using target sampling where points are drawn from the pool of geographic locations where we had sampled locality data [47].

For model evaluation we split the observed data into 80% training data and 20% testing data and computed area under the curve (AUC) and True Skill Statistic (TSS) as a measures of goodness of fit [48,49]. This split was performed once for each model for each species. To create an ensemble model per species, we combined models that had an AUC score greater than 0.75. We combined models for each species by calculating the mean habitat suitability predicted by each model.

### Factors influencing dispersal

We evaluated dispersal limitation using both an elevation-weighted cost distance metric to infer the current potential for dispersal between localities and time-since-divergence to evaluate the influence of time for dispersal between current localities given historical isolation (i.e., allopatric speciation). Cost distance is an effective predictor of dispersal limitation and population genetic structure, and is a better predictor of hummingbird beta-diversity between localities than Euclidean distance [27]. Computing cost distances among locations requires constructing an environmentally-weighted cost surface based on change in elevation, such that points in close proximity, but separated by large mountains or deep valleys, are more costly to cross than points separated by flat terrain. This model of dispersal incorporates both physical geographic barriers, as well as environmental change, since large changes in elevation also result in drastic changes in temperature and precipitation regimes. For each assemblage we generated a unique cost distance surface based on the elevation of that assemblage and calculated the least cost path from the focal assemblage to all other assemblages. This metric should not be seen as a predicted dispersal path, but rather a quantitative measure of geographic connectedness. Cost paths were calculated using the R package gDistance [50] using the mean transition probability of moving from one cell to any of its eight neighboring cell.

Next, for each pair of sister taxa (n = 58), we evaluated the relationship between time since divergence and the mean cost distance between their localities. As described above we used the change in elevation cost surface to calculate the least cost path between each presence locality of one sister taxa to the closest presence locality of the other sister taxa. Assemblages where both taxa co-occur had zero cost distance. We then modeled cost distance between sister taxa and modeled it as a function of time since divergence using linear regression. Given that there are relatively few early branching splits in the hummingbird phylogeny (only 3 splits greater than 10myr) and older splits could present undue leverage, we also evaluated the relationship solely among sister taxa younger than 10 myr.

### Biologically motivated species pools

To provide a null hypothesis for the observed pattern of phylogenetic relatedness in the hummingbird assemblages we used biologically motivated species pools. We created two types of null assemblages: environment assemblages and environment + dispersal assemblages. Environment assemblages were based on predicted habitat suitability from bioclimatic models for each species at each geographic location for which we had observed assemblage data. Defining predicted suitable areas required turning the index of suitability returned from the bioclimatic into a statement of predicted presence or absence. Due to differences in species prevalence, taking a fixed probability cutoff across all species would bias presence towards more common species [51]. We therefore applied a threshold to the suitability values for each species based on the distribution of suitability values from that species’ observed localities [45,52]. We used the probability of suitability that allowed 95% (quantile 0.05) of the known presences to be predicted present. The 0.05 quantile threshold maximized the number of true predicted presences in the observed data, while minimizing the number of false negatives.

In the environment + dispersal assemblages, predicted suitable locations were considered unavailable for species’ presence if the cost distance to the closest observed location was higher than a species-specific threshold. This threshold was determined by calculating the cost distance for every observed locality of that species to the closest observed locality. To avoid letting a single assemblage drive this value, we used the 95^th^ quantile of the distribution as the maximum cost distance between suitable sites.

### Statistical analysis

For each of the four simulations, the two predicted assemblages, and the observed data, we compared the relationship between phylogenetic distance to the closest related species and the probability of co-occurrence. The full model reads: the presence of hummingbird species (i) at site (Y) is a Bernoulli trial with a probability of occurrence *ρ*. The probability of occurrence is a logit transformed function of a species intercept (*α*), the linear effect of relatedness on occurrence (*β*_*i*,1_) and the non-linear effect of relatedness on occurrence (*β*_*i*,2_). We included a polynomial term because we believe multiple mechanisms may shape co-occurrence, leading to a non-linear relationship. We chose a hierarchical model that first fits a distribution to all species responding to the presence of closely related species (‘group level distribution’ ($\mu_{\beta_{1}}$), but then allows each species ($\sigma_{\beta_{1}}^{2}$) its individual response to relatedness (*β*_*i*_).

$$Y_{i}∼Bernoulli(\rho_{i})$$

$$logit(\rho_{i})=\alpha_{i}+\beta_{i}*x+\beta_{i,2}*x^{2}$$

$$\alpha_{i}∼Normal(\mu_{\alpha },\sigma_{\alpha }^{2})$$

$$\beta_{i}∼Normal(\mu_{\beta_{1}},\sigma_{\beta_{1}}^{2})$$

$$\beta_{2,i}∼Normal(\mu_{\beta_{2}},\sigma_{\beta_{2}}^{2})$$

All model parameters had non-informative priors (*Normal*(*mean* = 0.001, *precision* = 0.001) with variances *Gamma*(*shape* = 0.001, *rate* = 0.001)). All posterior probabilities for model parameters were estimated using Markov chain Monte Carlo (MCMC) in JAGS. Models were run for 50,000 draws with two chains, thinning by five draws.

## Results

**Fig 2:**
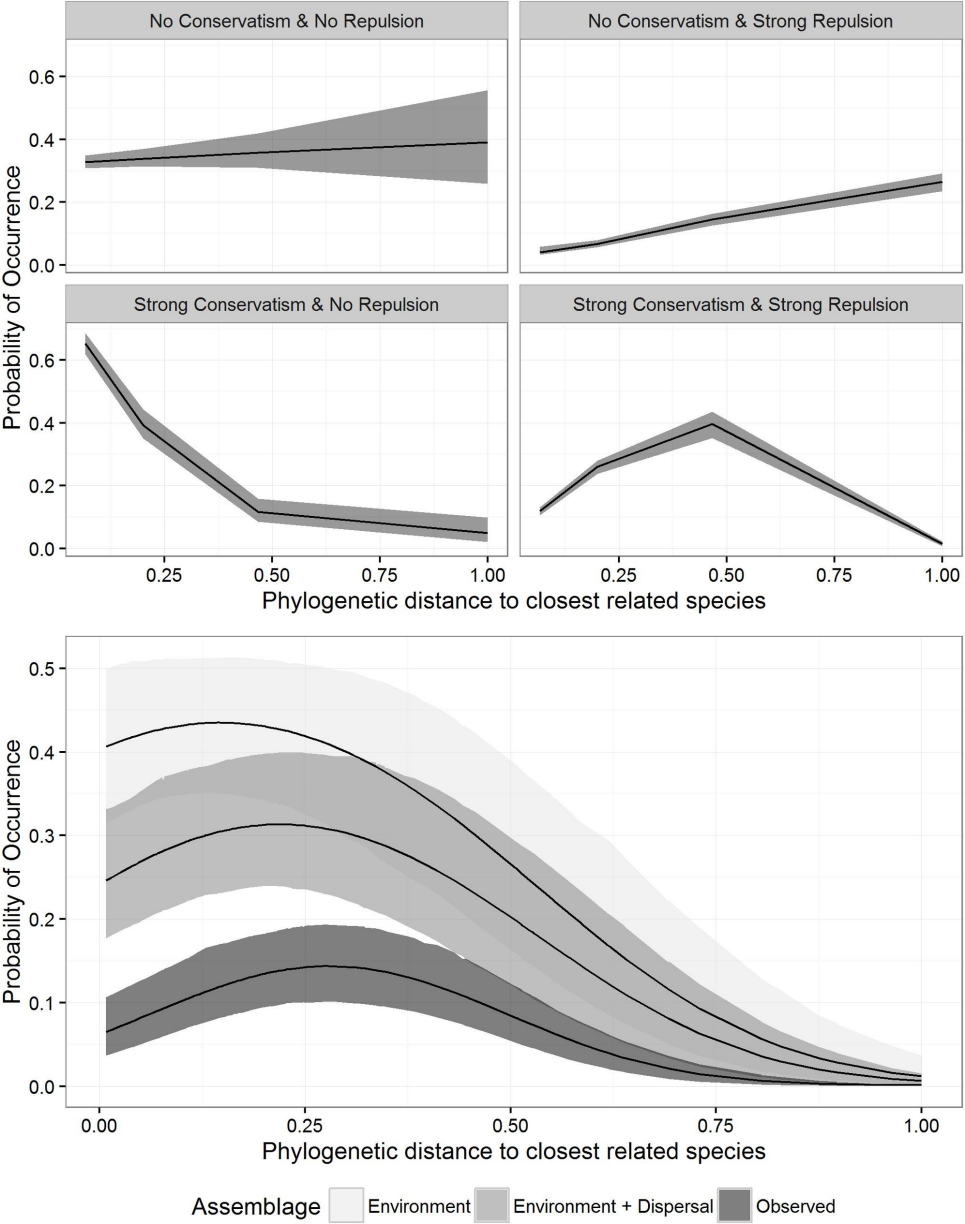
Probability of occurrence based on a thousand draws from the posterior distributions for each of the simulation assemblages (A) and for the observed and predicted assemblages (B). The simulated assemblages modeled hypothetical species occurrence based on a known model of trait evolution incorporating phylogenetic conservatism and repulsion among closely related species. The predicted assemblages are rearrangements of the true observed assemblages based on predicted habitat suitability (environment assemblages) and the addition of a dispersal filter (environment + dispersal assemblages). The hierarchical posteriors were plotted on the scale of the input data to show the estimated relationship between occurrence and the phylogenetic distance to the closest related species. The shaded region is the central 95^th^ quantile of the posterior distribution. The observed pattern of co-occurrence at first increases with increasing relatedness to a co-occurring species and then decreases in co-occurrence when a very closely related species is present. The overall observed co-occurrence is less than expected given species abiotic tolerances and potential dispersal.

Our simulations showed that as phylogenetic conservatism in environmental tolerances increased, species were more likely to co-occur with closely-related species (Fig 2). When environmental tolerances evolved without either phylogenetic conservatism or repulsion, relatedness had little impact on co-occurrence. When environmental tolerances evolved without phylogenetic conservatism, but with phylogenetic repulsion, there was a strong positive relationship between distance to the closest related species and presence. Finally, when the occurrence trait evolved with both phylogenetic conservatism and repulsion, there was increasing co-occurrence when a moderately related species was present, followed by a decrease in co-occurrence when a closely related species was present.

To create biologically meaningful null models of assemblage structure, we used bioclimatic models and cost path analysis to create predicted assemblage lists based on predicted habitat suitability and dispersal limitation. For each of the 132 species in the observed assemblage lists, we created an ensemble model from each of the GLM, MAXENT, and GBM models that had an AUC score greater than 0.75. This yielded a final count of 100 species occurring in 230 geographic assemblages. The AUC of these models was high across all individual model types (mean: GBM = 0.97, GLM = 0.87, MAXENT = 0.90). Although TSS scores were more variable across modeling types (mean: GBM = 0.87, GLM = 0.65, MAXENT = 0.65), the correlation between AUC and TSS scores was high across all models and species (GBM = .0.97, GLM = 0.96, MAXENT = 0.91), suggesting that our evaluation of model performance was consistent across metrics. Twenty-five of the 32 species excluded from the observed assemblage lists occurred in less than 10 localities, and five species occurred in just a single locality. On average observed assemblage lists had 91% completeness, and in 121 of the 230 assemblage lists all species were corrected predicted as present. We therefore limited our analysis of co-occurrence to species shared by all observed, environment and environment + dispersal assemblages.

**Fig 3:**
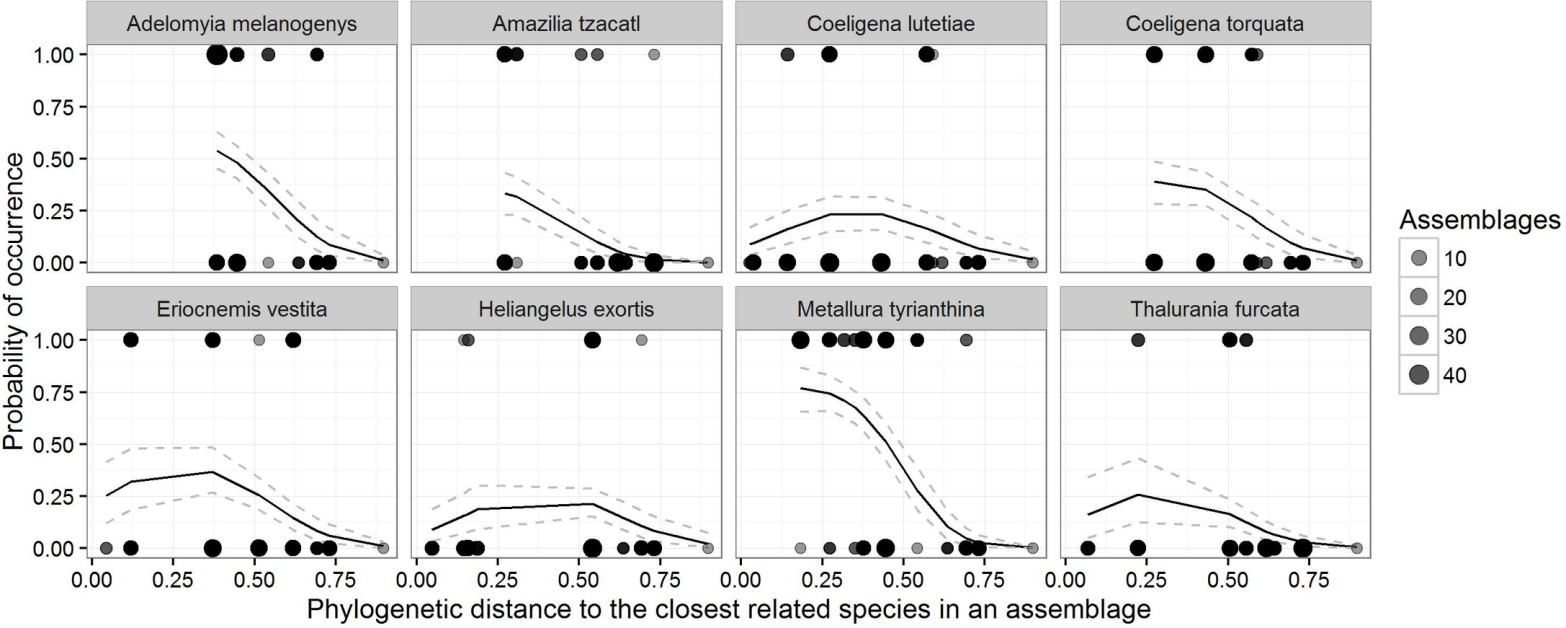
Example species level curves for probability of occurrence and phylogenetic distance to the closest related species. The graph shows the presence (y = 1) or absence (y = 0) of a given species at each of the geographic assemblages.Since the nearest closest related species are often the same species in multiple assemblages, there are multiple points overlapping. The number of assemblage lists that define each point is shown by the size of the point. Species were selected to show a variety of co-occurrence curves and represent a diversity of phylogenetic lineages within the hummingbird clade.

In both the environment and environment + dispersal assemblages, the probability of a species’ occurrence initially increased with decreasing phylogenetic distance to a species in an assemblage, followed by a decrease among closely-related species. The environment + dispersal assemblages showed a lower co-occurrence than environment suggesting that dispersal between localities was a factor in shaping predicted species lists. We compared the environment and environment + dispersal assemblages to our observed assemblage data to determine whether we would have arrived at the observed pattern based solely on environmental tolerances. Similar to the predicted assemblages, the observed assemblages showed an initial increase in the probability of co-occurrence with increasing relatedness, followed by decreased co-occurrence among closely related species (Fig 2). Explorations of species-specific responses showed that data-poor species had wider credible intervals, and our observed pattern is present in many, but not all, species throughout the hummingbird phylogeny (Fig 3). Given the lack of overlap in the confidence intervals of the estimate among the observed and environment assemblages, we infer that co-occurrence is less than expected among closely related species. The confidence interval of the observed and environment + dispersal assemblages had minor overlap, suggesting that including a dispersal filter reduced co-occurrence of related species.

**Fig 4:**
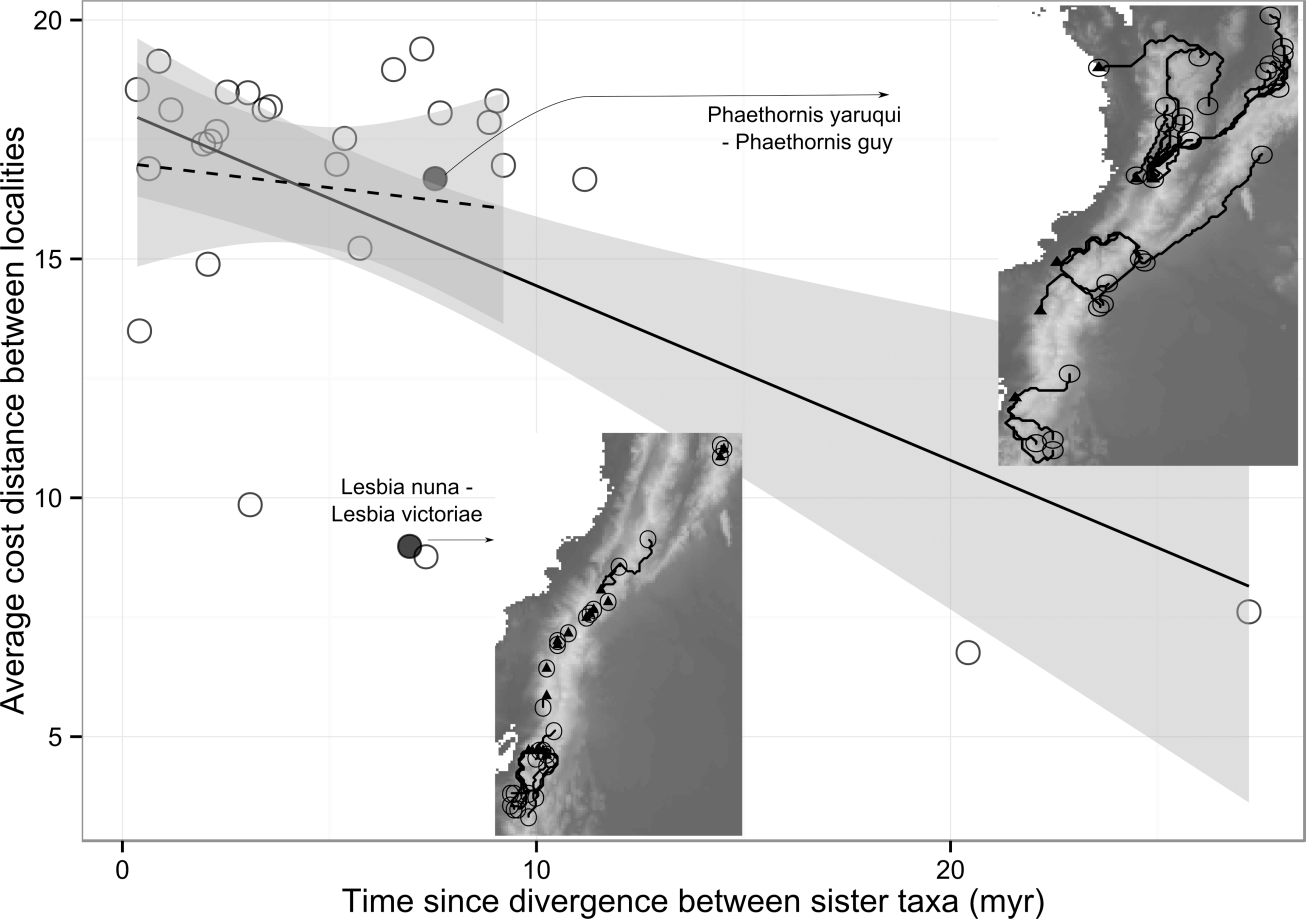
Average cost distance between localities for each pair of sister taxa. The cost distances incorporates the change in elevation and Euclidean distance as a measure of geographic barriers between localities. We calculated the average cost distance between each presence locality of one sister taxa to the closest presence locality of the other sister taxa. Assemblages where both taxa co-occur had zero cost distance. The solid regression line is for all species pairs, and the dashed regression line is for only species pairs that diverged less than 10 myr ago. Two example sister pairs are shown. The *Phaethornis* sister taxa have high cost distance due to presence of the Andes, whereas the *Lesbia* sister taxa have relatively low cost distance due to their short distance along mountain slopes. In the insets, one sister taxa is shown as a solid triangle, and the other an open circle. The least cost path along the change in elevation frictional surface is shown in black.

To estimate the importance of time on the dispersal on co-occurrence of closely species, we looked at the relationship between time since divergence and the presence of geographic barriers (Fig 4). We found that older sister taxa were separated by smaller changes of elevation, but the relationship was only significant when including the two deepest sister taxa pairs (R^2^ = 0.32, df = 27, p = 0.01). For species pairs less than 10 myr, there was only a modest, non-significant, relationship between the time since divergence and average cost distance between localities of sister taxa (R^2^ = 0.01, df = 24, p = 0.6).

## Discussion

The relative roles of environmental filtering, dispersal, and competition in promoting the co-occurrence of related species remains a central focus of biogeography. We found the relationship between co-occurrence and relatedness was non-linear; the probability of co-occurrence was greatest for moderately related species and decreased among both distantly and closely related species. The increase in co-occurrence among moderately related species as compared to distantly related species is likely due to environmental filtering based on shared environmental tolerances.

The pattern of reduced co-occurrence among closely-related species could be caused by interspecific competition, or allopatric speciation with insufficient time for re-expansion to secondary sympatry [21,36,53]. Because reduced co-occurrence was maintained when predicted suitable environments and dispersal filters were considered, our results suggest that interspecific competition may be a factor shaping distributions at a broad spatial scale [53–55]. While these results are not conclusive proof that competition influences range dynamics and community structure, they present an interesting hypothesis worth investigating at local scales [56]. For example, future studies could conduct local scale observations or experiments to evaluate if resource competition increases among closely related species [14].

Environmental filtering, which results in co-occurrence of related species with similar traits, is well documented across multiple taxonomic groups [57], including hummingbirds [23]. In hummingbirds, only some lineages, notably the brilliants and coquettes, are adapted to high-elevation environments [22,58,59]. Species in these lineages have adapted physiological and biomechanical traits needed to thermoregulate in low temperatures, and sustain flight in low partial pressures [60]. For example, several high-elevation lineages have amino acid substitutions associated with hemoglobin adaptation to low oxygen environments [61]. In contrast, *Phaethornis* hermits are widespread throughout the lowlands, but their elevation ranges rarely extend into the highlands [62].

Hummingbirds compete for nectar resources and related species often use similar resources [63,64]. Biogeographic analyses of hummingbird-plant networks show that related species tend to have similar levels of niche breadth and specialization [65]. Historical constraints, either through phylogenetic tradeoffs or past climate influences may lead to constraints on foraging breadth and increased overlap among related species[66,67]. At a local scale, competition among hummingbird species appears to be mediated by morphological traits, such as bill length, that determine the degree of resource overlap [68,69]. Given that hummingbird traits exhibit weak trait lability [70], then closely related species may compete more for limited floral resources [22]. This mechanism would result in the lack of co-occurrence of closely related species at the biogeographic scale.

Biogeographic patterns suggestive of competition have been reported in several other groups [45,54]. Sylvia warblers with greater dispersal ability occupy a greater proportion of potential suitable habitat, especially where there are few congeneric species [55]. Similarly, *Anolis* lizards are less likely to occur in predicted suitable habitat when a morphologically similar species is present [53]. Recent physiological measurements of basal metabolic rate showed little variation across tropical birds along a wide altitudinal gradient [71], suggesting that biotic interactions may have a role in setting altitudinal range limits [72]. In addition, comparisons of the elevation ranges between the main Andes and less diverse isolated mountains showed that some birds and mammals have broader elevational ranges on isolated mountains [45,73]. These patterns were interpreted as potential evidence for competition limiting elevation ranges, but bioclimatic models suggest that in birds, they were at least partly due to differences in environment among similar elevation ranges on different mountain slopes [74]. In our case, we explicitly evaluated whether the lack of co-occurrence is due to environment by comparing our observed assemblages to those predicted based on bioclimatic models.

To investigate whether dispersal explains the lack of co-occurrence among very close relatives we tested the relationship between sister taxa and cost distance as a proxy for dispersal barriers. We found some evidence that older sister taxa are, on average, separated by smaller geographic barriers and tend to co-occur more frequently than younger sister taxa. This result could be interpreted as incomplete range filling [8], since allopatric speciation is common in hummingbirds [75,76]. Macroecological analysis of primates and songbirds indicate that the rate of range overlap of sister taxa increases as a function of time since divergence [21,77]. However, our data only show this pattern when including the oldest lowland sister divergences (2 sister taxa comparisons are greater than 10myr). Excluding these two taxa, we do not see any relationship for the rest of the hummingbird sister taxa (23 sister taxa comparisons are less than 10myr).

There are several reasons why we hesitate to ascribe the pattern of reduced co-occurrence of closely related species wholly to insufficient time for dispersal. If dispersal limitation over evolutionary time was a driving force in community assembly, we would expect clades to be highly localized within areas surrounded by geographic barriers of unsuitable habitat [78]. Comparisons across barriers should then show high phylogenetic beta diversity, but low phylogenetic alpha diversity [79]. However, when comparing lowland assemblages across the Andes we observed the opposite pattern with relatively low phylogenetic beta diversity and high phylogenetic alpha diversity [27]. In addition, we would expect younger clades to have a smaller extent of occurrence compared to older clades, since there has been less time to occupy potentially suitable habitat. Yet, the youngest hummingbird clade, the Bees, are less than 2.5 million years old and range from Alaska to the Tropics. Hummingbirds are strong fliers and many species make latitudinal or elevational migrations with well-established patterns of vagrancy. For instance, the American Ornithology checklist has records for 23 hummingbird species, even though only 10 species commonly breed north of the US-Mexico border [80]. Together, these lines of inference suggest that dispersal is unlikely to be the sole mechanism in occupying potentially suitable habitat.

The role of niche conservatism in promoting co-occurrence of related species through environmental filtering is well documented [57]. The more challenging question is whether the reduced co-occurrence of close relatives is related to dispersal limitation or competition [12]. Ultimately, these two factors are conflated in macroecological analysis, since the species for which we expect the greatest ecological overlap are the same species for which there has been less time since divergence. We hope that our approach, which considers the simultaneous effects of multiple mechanisms on biogeographic patterns of co-occurrence, will serve both as a model for similar biogeographic studies and a stimulus for more detailed local investigation.
